# Amelioration of Physical Properties and Printability of Paper Coated with N-methylated Chitosan

**DOI:** 10.1038/s41598-020-66827-8

**Published:** 2020-06-18

**Authors:** Meiyan Wu, Rui Xu, Chao Liu, Bin Li, Zhu Long

**Affiliations:** 1 CAS Kay Laboratory of Biofuels, Dalian National Laboratory for Clean Energy, Qingdao Institute of Bioenergy and Bioprocess Technology, Chinese Academy of Sciences, Qingdao, 266101 China; 2 Key Laboratory of Eco-textiles, Ministry of Education, Jiangnan University, Wuxi, 214122 China; 3 Key Laboratory of Pulp and Paper Science & Technology of Ministry of Education/Shandong Province, Qilu University of Technology (Shandong Academy of Sciences), Jinan, 250353 China

**Keywords:** Carbohydrate chemistry, Polysaccharides

## Abstract

In offset printing process, poor mechanical properties and printability of paper substrate usually result in printing problems, low quality of print and waste of paper materials. Therefore, many researches focus on the quality improvement of paper substrates using suitable additives. In this work, N-methylated chitosan, including N, N-dimethyl chitosan (DMC) and N, N, N-trimethyl chitosan (TMC), were prepared and employed as coating agents to ameliorate the mechanical properties and printability of paper sheets. Analysis results showed that the mechanical strength of coated papers with DMC and TMC were largely improved, because the fibers with negative charges were prone to form electrostatic bonding with the positively charged N-methylated chitosan, thus enhancing paper strength. Particularly, compared with chitosan and DMC, the TMC-coated paper exhibited better mechanical properties, printability and surface properties due to the high cationic charge density of TMC. Therefore, surface coating with TMC is of great benefit to decrease the printing problem of paper sheets and enhance the operation speed in offset printing. This work provides a valuable reference for the amelioration of the printability and physical properties of high-quality paper products for many promising applications.

## Introduction

Up to now, paper sheet made by lignocellulosic fibers is one of the most widely used materials in the world due to its good recyclability and biodegradability^1^. However, the mechanical strength and printability of paper still need to be improved to reduce the printing problem in offset, such as paper breaking, picking telegraphing, and poor color reduction^2^. To overcome these issues, one effective method is to coat polymer onto paper sheet. Starch^3,4^, chitosan^5,6^, beeswax^7^, soy protein^8^ and polyvinyl alcohol (PVA)^9^ have stimulated considerable interest as high-efficiency coating agents. Among them, chitosan with the structure of (1–4)-linked 2-acetamido-2-deoxy-d-glucopyranose is the only positively charged natural biodegradable polymer, which is fully or partially deacetylated chitin^10^. The protonation of amino groups in chitosan under acidic conditions leads to the formation of positively charged surface of chitosan^11^. In papermaking process, the bonding between the fibers of paper can be increased by the electrostatic adsorption between the positive charge of amino groups on chitosan chain and the negative charge on the surface of fiber in water^4,12,13^. Therefore, chitosan can be used as a wet-end additive to increase the strength of paper. Moreover, chitosan with good film-forming and antibacterial properties is a suitable cationic polymer as a coating material^14^. The strength and the barrier properties against water vapor and oxygen of paper substrates can be largely increased after coated by chitosan^6,15^.

However, chitosan is insoluble in neutral and alkaline conditions due to the amino group in chitosan with a pKa value of 6.5, which limits its application. N-methylation is a way to directly modify chitosan by introducing alkyl groups onto N site of the structure of chitosan. It has been demonstrated that the hydrogen bonding of N-H was reduced and the positive charge of chitosan in an acid solution was increased after H atoms on N substituted by methyl^16,17^. N, N-dimethyl chitosan (DMC) and N, N, N-trimethyl chitosan (TMC) are N-methylated chitosan by introducing two and three methyl groups onto N site of chitosan, respectively. The chemical modification of chitosan can reduce the rigid structure of chitosan, hence improving its solubility for end applications. According to the previous reports, there are three kinds of N-methylation agents to produce TMC, including dimethyl sulfate, methyl iodide and dimethyl carbonate^18–20^. Methyl iodide is a carcinogenic and very expensive solvent, which is generally used in the high value-added filed^21^, such as pharmaceutical engineering, analytical chemistry, organic synthesis, *etc*. Moreover, although dimethyl carbonate was reported as a green solvent, the reaction of dimethyl carbonate usually requires high temperature and high pressure due to the low reactivity^22^. In our previous work, TMC was prepared using dimethyl carbonate^20^. The preparation process of TMC was firstly conducted to produce dimethyl chitosan (DMC), and then DMC in an ionic liquid was reacted with dimethyl carbonate under high temperature (150 °C). Compared with dimethyl carbonate, dimethyl sulfate can be used to directly produce TMC from chitosan in an alkaline solution without the assisting of ionic liquid, and the required temperature for the reaction is relatively lower (room temperature to 70 °C)^18^. Thus, this low-priced one step approach using dimethyl sulfate as a N-methylation agent was employed to prepare TMC in this work.

Nowadays, TMC, a soluble quaternary ammonium salt of chitosan, is mostly used as mucosal permeation enhancers^23,24^, nonviral vectors^25^, and antimicrobial agent^26^. In addition, it has also been reported that TMC nanoparticles could be attached to viscose cellulose fiber as a potential drug delivery system^27^, and TMC was also applied onto cotton fabric by the pad-dry cure method for progressively improving the absorbency, dyeing behavior and wrinkle recovery^28^.

Therefore, it is expected that both DMC and TMC have great potential to be used as treatment agents for paper sheets, which are also formed by cellulose. In the present work, the effect of N-methylated chitosan on the mechanical properties, printability and surface property of the coated paper was comprehensively investigated. The results will be of great importance for the amelioration of printing efficiency and end paper quality, particularly for the high-quality packaging and decoration applications.

## Results and discussion

### Structure and water solubility analysis of methylated chitosan

**Figure 1:**
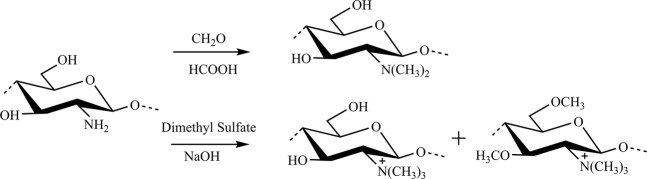
Synthesis routes of DMC (up) and TMC (down).

**Figure 2:**
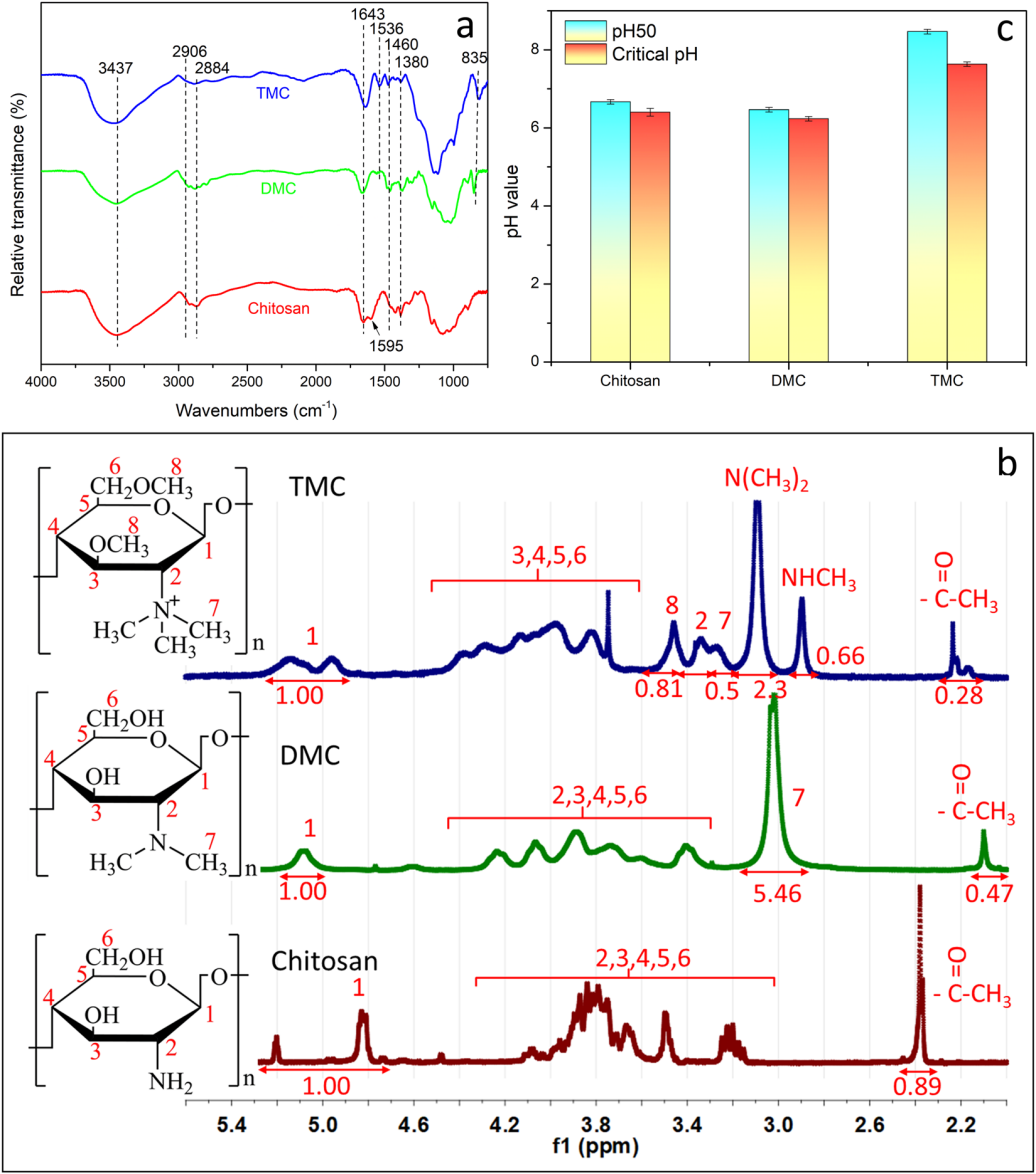
Structure and water solubility analysis of N-methylated chitosan. (**a**) FTIR spectra of chitosan and N-methylated chitosan. (**b**) ^1^H NMR spectra of chitosan and N-methylated chitosan. (**c**) Solubility of chitosan and N-methylated chitosan.

DMC and TMC were prepared by the reaction of the methylation reaction, and the synthesis routes of DMC and TMC are shown in Fig. 1. Then, the structure of chitosan and N-methylated chitosan (DMC and TMC) was firstly investigated by FTIR spectroscopy. As shown in Fig. 2a, the broad peak around 3437 cm^−1^ belongs to the stretching vibrations of O-H and N-H bonds, and the peaks at 2906 and 2884 cm^−1^ are attributed to the asymmetrical stretching C-H bonds^29^, which is observed in all spectra. Moreover, the peak at 1380 cm^−1^ is assigned to C-N bonds in the structure of chitosan^15^. In addition, the two peaks at 1643 cm^−1^ and 1595 cm^−1^ in the spectrum of chitosan belong to amide I and amide II, respectively. Amide I mainly derives from the C = O bonds vibrations of the amide and amide II is mainly dependent on the angular deformation of N-H bending^20^. Compared to chitosan, there are two new peaks at 1536 cm^−1^ and 1460 cm^−1^ caused by CH_2_ and CH_3_ for DMC and TMC. Simultaneously, the peak at 1595 cm^−1^ assigning to N-H bonds disappears in the spectra of DMC and TMC^30^. Simultaneously, there is a new peak at 835 cm^−1^ in the spectra of methylated chitosan due to C-H out-of-plane deformation^31^. These phenomena indicate that methyl groups are introduced to the amino structure of chitosan after methylation reaction^32,33^.

^1^H NMR spectra of chitosan and methylated chitosan were tested for further characterization, as shown in Fig. 2b. From the spectrum of chitosan, the signal between 4.75 and 5.25 ppm is attributed to hydrogen bonded to the anomeric carbon 1^34^, the signals between 3.10 and 4.15 ppm corresponds to hydrogen atoms of the glucopyranose ring, and the signal at 2.35 ppm is assigned as hydrogen atoms of the acetyl group^35^. Compared to chitosan, there is a new signal at 3.0 ppm in the spectrum of DMC, which belongs to hydrogen atoms of dimethyl group^20^. In the spectrum of TMC, the new signals can be observed at 2.9 and 3.1 ppm, which are attributed to the hydrogen atoms of methyl groups and dimethyl groups^36^, respectively. Furthermore, the TMC spectrum shows the signals at 3.25 ppm and 3.45 ppm, which are assigned to hydrogen atoms of trimethyl groups and methoxy group (-OCH_3_), bonded to carbon 3 and carbon 6^18^, respectively. This result is due to the weak selectivity of dimethyl sulfate to NH_2_ and OH groups of chitosan in the process of methylation^37^. Therefore, the structure of DMC and TMC were proved by ^1^H NMR and FTIR results.

In addition, the degree of substitution (DS) of DMC and the degree of quaternization (DQ) of TMC can be calculated by the integral area of the hydrogen atoms from the ^1^H NMR spectrum according to formula (1) and formula (2), respectively. Meanwhile, the degree of O-methylation (DO) of TMC can also be calculated with the same method. According to the calculation of formulas (1) and (2), the DS of DMC was 91.0%, while the DQ and DO of TMC were 5.56% and 13.5%, respectively.

1 $${\rm{DS}}=\frac{\frac{{I}_{DMC}}{6}}{{I}_{H1}}\times 100 \% $$

Where *I*_*DMC*_ is the integral of the hydrogen atoms on dimethyl group, and *I*_*H1*_ is the integral of the hydrogen atoms bonded to Carbon 1^20^.

2 $${\rm{DQ}}=\frac{\frac{{I}_{TMC}}{9}}{{I}_{H1}}\times 100 \% $$

Where *I*_*TMC*_ is the integral of the hydrogen atoms on the quaternary ammonium group.

The water solubility results of N-methylated chitosan are shown in Fig. 2c. The 50% of transmission rate of chitosan solution (pH50) is at the pH of 6.7, while the 98% of transmittance rate of chitosan solution (Critical pH) is at the pH of 6.4. Compared with chitosan, the solubility of DMC has no obvious change, but the pH50 and Critical pH of TMC are 8.5 and 7.6, respectively. This result suggests that the solubility of chitosan derivative with quaternary ammonium can be greatly improved^38^, and it can be dissolved in the pH range from 1 to 7.6 at the concentration of 2 mg/mL, which effectively expand the scope of solubility. Good water solubility of TMC under neutral conditions is of great help to reduce the equipment corrosion and the loss of paper strength by acid solution, thus reducing the production cost of high-quality paper sheets.

### Mechanical properties of coated papers

As known, the mechanical properties of paper sheets are of great importance for the manufacture and printing process^39,40^. Therefore, the mechanical properties of coated papers, including tensile index, burst index, tearing index and wet tensile index, were tested to evaluate the effect of coating with N-methylated chitosan. As reported, the mechanical properties of paper sheets depend on the quality of fiber itself and the bonding strength between fibers^41,42^. The bonding strength between fibers can be increased by the supplement of additives in pulp^43^ or surface coating on paper sheets^9^.

**Figure 3:**
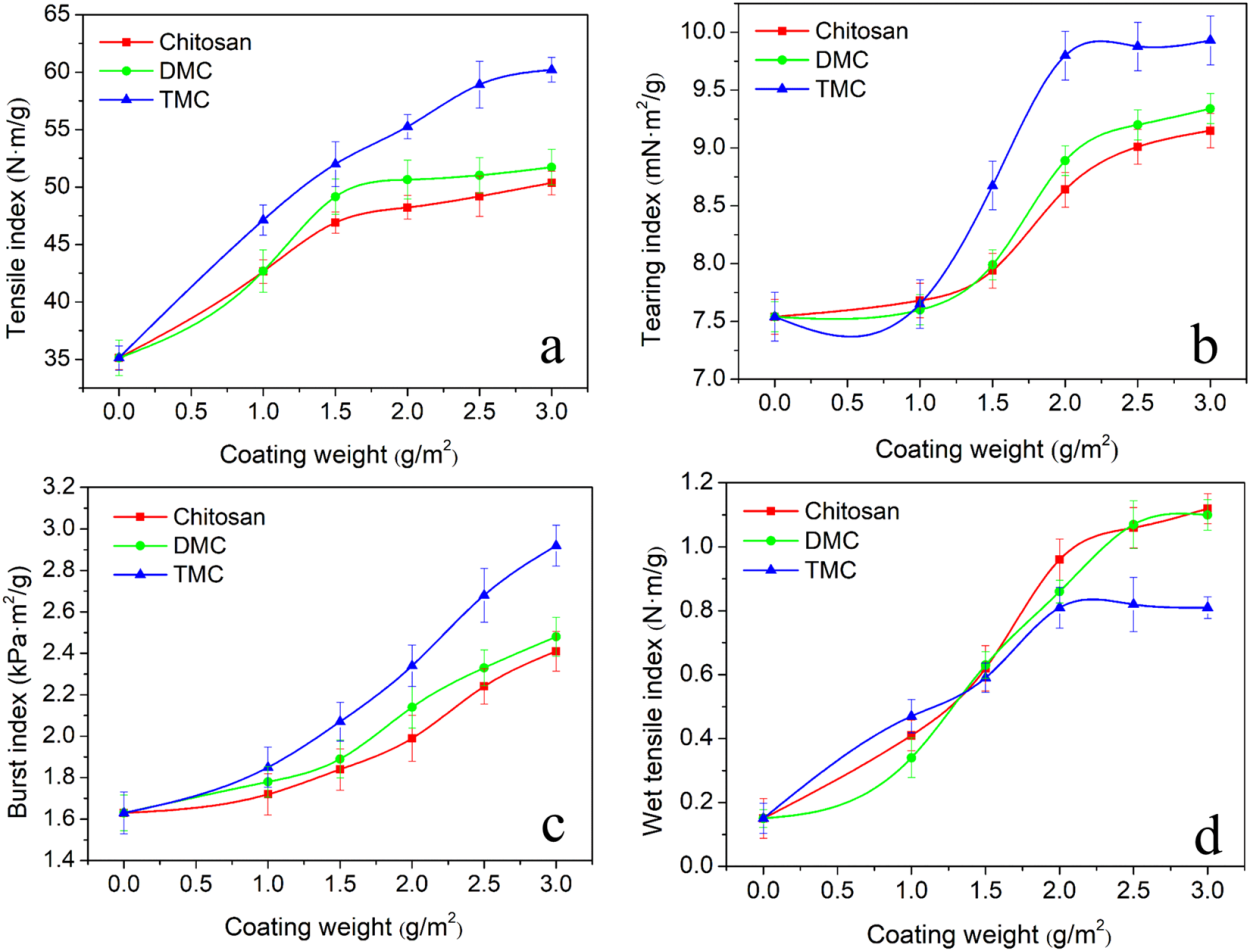
Mechanical properties of coated papers. (**a**) Tensile index. **(b)** Tearing index. (**c**) Burst index. **(d)** Wet tensile index.

As shown in Fig. 3a, the tensile index of paper sheets is largely increased with the increase of coating weight. Especially, the tensile index of TMC-coated paper increased from 35.1 to 60.2 N·m/g with the coating weight of 3.0 g/m^2^, which is increased by 71.5%. Meanwhile, the trend of the tearing index of paper sheet coated with methylated chitosan is similar with the tensile index of coated paper, and the tearing index of chitosan, DMC and TMC coated paper with the coating weight of 3.0 g/m^2^ is increased by 21.4%, 23.9%, 31.7%, respectively (Fig. 3b). Moreover, Fig. 3c shows that the growth rate of the burst index is slow with a lower coating weight of methylated chitosan (<1.5 g/m^2^), but there is a rapid increase of the burst index with the coating weight higher than 1.5 g/m^2^. When the coating weight is the maximum dose of 3.0 g/m^2^, the burst index of TMC-coated paper also arrives at maximum, which is increased by 79.1% compared with the control. In addition, when the coated weight is 3.0 g/m^2^, the wet strength of paper sheets coated with chitosan, DMC and TMC increased 6.5, 6.3 and 4.4 times, respectively (Fig. 3d).

**Figure 4:**
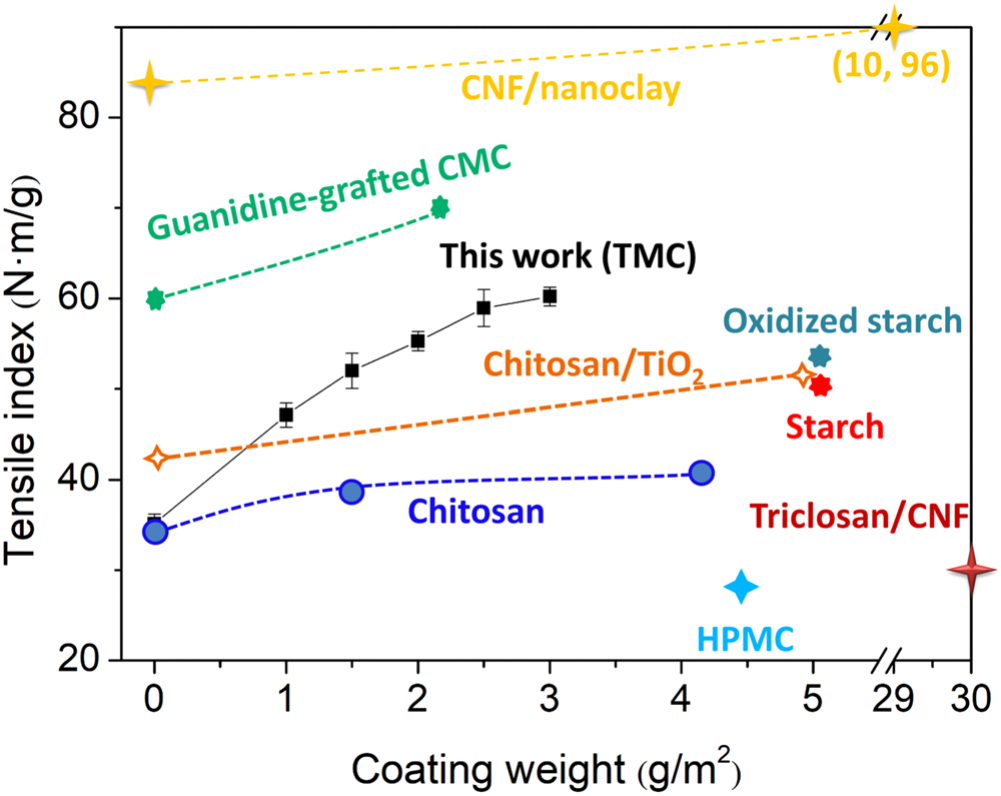
Comparison of the tensile index of the coated papers with different coating reagents. Note: Chitosan^63^, Starch and oxidized starch^44^, Chitosan/caseinate^6^, Chitosan/TiO_2_^45^, Triclosan/CNF (Cellulose nanofibrils)^46^, HPMC (Hydroxypropyl methylcellulose)^47^, CNF/nanoclay^48^, guanidine-grafted CMC (Carboxymethyl cellulose)^49^.

In addition, the tensile index of coated paper with TMC (this work) is compared with that of coated papers with other typical regents in Fig. 4. As can be seen, the tensile index of the coated paper with TMC is higher than that of the coated papers with starch^44^, oxidized starch^44^, chitosan/caseinate^6^, chitosan/TiO_2_^45^, triclosan/CNF (cellulose nanofibrils)^46^ or HPMC (hydroxypropyl methylcellulose)^47^, and lower than that of the coated papers with CNF/nanoclay^48^, and guanidine-grafted CMC (carboxymethyl cellulose)^49^. However, although the used paper substrate had a lower tensile index, the increasing rate (71.5%) of tensile index for the TMC-coated paper (with coating weight of 3.0 g/m^2^) was clearly higher than the CNF/nanoclay (14.3%, coating weight 10.0 g/m^2^) and guanidine-grafted CMC coated papers (16.7%, coating weight 2.1 g/m^2^). Therefore, TMC is superior to some commonly used coating agents, such as starch, HPMC, for improving the mechanical strength of the coated paper.

To investigate the mechanism of the improvement of paper strength, the ionic charge density of different additives was tested accordingly. The ionic charge density of additives with the concentration of 0.1 wt.% in acid solution are illustrated in Table 1. It was measured under pH value of 3, 5 and 7 in consideration of acid soluble chitosan. As expected, the cationic charge density of TMC solution is higher than that of chitosan and DMC solutions under acid and neutral conditions. Especially, with pH value of 3 and 5, the cationic charge density of TMC solution is increased by 34.6% and 33.8%, respectively, in comparison with chitosan. The fibers with the negative charges are prone to form electrostatic bonding with the cationic charged N-methylated chitosan with the increase of introducing methyl groups onto amino group of chitosan^50^. This effect leads to the enhancement of the adhesion strength of the fibers^51^. Therefore, TMC is a suitable and effective dry strengthening agent to improve the mechanical properties of paper sheet. Yet, chitosan coating yields a better wet tensile strength of coated paper in comparison with methylated chitosan (Fig. 3d), which is probably due to the insolubility of chitosan in water^15^.

**Table 1:** Cationic charge density of chitosan and N-methylated chitosan.

pH value	Cationic charge density (meq/g)		
	Chitosan	DMC	TMC
pH = 3	0.205 ± 0.002	0.234 ± 0.002	0.276 ± 0.001
pH = 5	0.201 ± 0.001	0.228 ± 0.003	0.269 ± 0.002
pH = 7	Not detected	0.053 ± 0.004	0.120 ± 0.002

### Printability of coated paper

Printability of paper sheet plays a decisive role in the quality of print. After coating on the surface of paper sheets, the coating properties of coated paper, such as the surface strength, ink absorbency and liquid absorbency, are the crucial factors affecting printability of paper sheet^52^. Therefore, it is important to investigate the effect of those factors on paper printability after coating with chitosan and N-methylated chitosan.

Surface strength of paper sheets refers to the bonding between filler or fiber fines and fiber materials on the surface of paper substrate^53^. Low surface strength of paper sheets can result in easy absorption between filler or fiber fines and ink, and then the picking and linting on the surface of paper sheets can be observed due to the tension of ink in printing process^54^. Surface strength of coated paper are exhibited in Table 2. After coated with chitosan and N-methylated chitosan, the surface strength of all paper samples was improved. In particular, when printing speed maintains 5.5 m/s, the surface strength of TMC-coated paper (3.44 m/s) is still higher than that (3 m/s) of chitosan-coated paper. These results suggest that effective adsorption happens between TMC and the surface of paper sheet, and TMC plays a bridge role between fibers to improve the degree of the combination of fibers^24^. Fiber fines and filler can firmly stay on the surface of paper sheets even at a high printing speed^55^. Thus, due to the increasing of the surface strength of papers by coating with TMC, the operation speed of printing press can be increased, which is of great importance for the improvement of production efficiency.

**Table 2:** Surface strength of coated papers.

Paper sample	Printing speed (m/s)	Surface strength (m/s)
Paper substrate	5	2.79 ± 0.03
Chitosan-coated paper	5	3.00 ± 0.02
DMC-coated paper	5	3.54 ± 0.03
TMC-coated paper	5.5	3.44 ± 0.03

Ink absorbency of coating is an important factor in printing. If the ink absorption of coating is too high, it can cause the gradual loss of color printing, the poor color reducibility, telegraphing and other issues. If it is too low, it can cause the printing problems of dot gain and smearing on the back^52^. In this work, the reflex factor of green ray before and after ink absorption was tested (Table 3), and K&N ink absorption value of coated papers with chitosan and N-methylated chitosan was calculated by the following formula (3).

3 $$Y=\frac{{Y}_{0}-{Y}_{1}}{{Y}_{0}}\times 100 \% $$

Where *Y* is the K&N ink absorption value, *Y*_0_ is the reflex factor of green ray and *Y*_1_ is the reflex factor of green ray after ink absorption.

**Table 3:** Ink absorption ability of coated papers.

Paper sample	*Y*_0_ (%)	*Y*_1_ (%)	*Y* (%)
Paper substrate	75.65 ± 0.01	41.67 ± 0.03	44.92
Chitosan-coated paper	73.15 ± 0.05	42.11 ± 0.06	42.43
DMC-coated paper	74.26 ± 0.03	34.96 ± 0.04	52.92
TMC-coated paper	76.90 ± 0.05	33.44 ± 0.05	56.51

It can be observed that when the coating weight is 1.5 g/m^2^, the ink absorption value of chitosan-coated paper is lower than that of paper substrate due to the good oil-resistance of chitosan^56^. However, compared with chitosan-coated paper, the coated papers with N-methylated chitosan exhibit applicable ink absorbency. This phenomenon is because N-methylated chitosan (DMC and TMC) coated papers have suitable smoothness for absorption ink in printing process. In addition, because a large amount of positive charges is equipped on the surface of TMC (Table 1), the ink binder made of the negatively charged resin or oil can be absorbed by positively charged TMC. Thus, ink can be firmly absorbed on the surface of TMC-coated paper. Therefore, TMC-coated papers have better ink absorbency for offset printing in comparison with chitosan and DMC.

**Figure 5:**
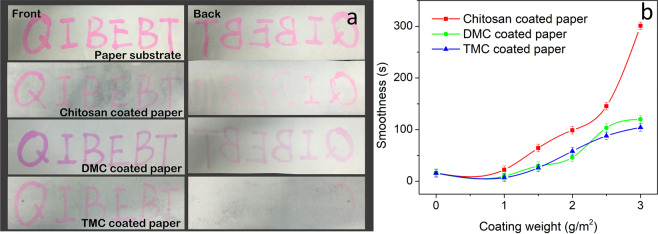
(**a**) Liquid absorbency of coated papers with chitosan and N-methylation chitosan. (**b**) Effect of coating weight on smoothness of coated paper.

In offset printing process, fountain solution should be first adsorbed on the paper, and then ink can be printed according to the principle of oil-water incompatibility^57^. As a result, paper sheet needs a certain liquid absorption to accelerate the permeation of fountain solution. However, excessive liquid absorption is easy to cause the printing trouble of telegraphing^58^. After coating with chitosan and N-methylated chitosan, the new forming film on the surface increases the barrier properties of paper and simultaneously decreases the liquid permeation. Liquid permeation and coating uniformity of coated paper can be measured according to the liquid permeation test. The front and back of coated papers after brushing with rhodamine B solution are photographed in Fig. 5a. Compared with paper substrate, the pale brushing imprint and less solution permeation of chitosan-coated paper can be seen. While DMC-coated paper not only exhibits clear brushing imprint and high color reduction in the front, but also shows low degree of permeation in the back. Particularly, the permeation degree of TMC-coated paper is even lower than that of DMC-coated paper. These results indicate that TMC-coated paper has a suitable liquid absorbency, and the printing problem of telegraphing is less likely to happen in the event of TMC as a coating agent.

### Surface properties of coated paper

**Figure 6:**
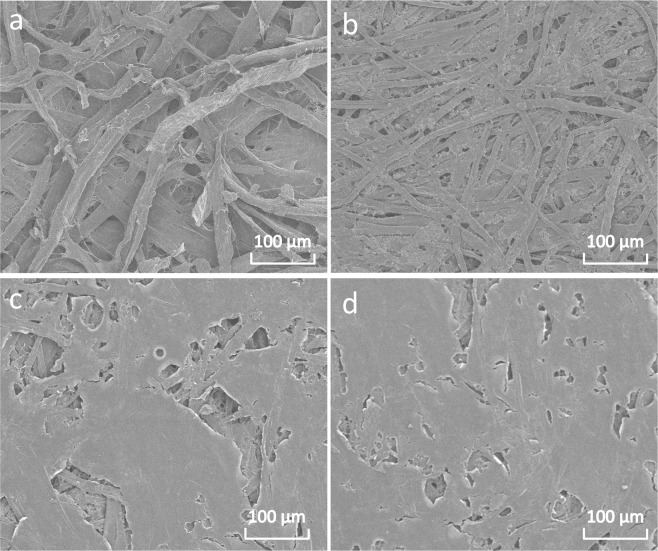
SEM images of coated papers. (**a**) Paper substrate. **(b)** Chitosan-coated paper. **(c)** DMC-coated paper. **(d)** TMC-coated paper.

Smoothness of paper refers to the leaking time of a certain volume of air going through between the smooth glass plate and the paper sheet under a certain vacuum degree. For those paper sheets which need to be printed, the printing density and the color reproducibility of coated papers are related to the smoothness of paper sheets. Smoothness of coated papers largely depends on the degree of coating uniformity^59^. The smoothness of coated paper with chitosan and N-methylated chitosan are given in Fig. 5b. When the coating weight is less than 1.0 g/m^2^, there is no obvious change in smoothness of coated paper with chitosan and N-methylated chitosan. However, when the coating weight is larger than 1.0 g/m^2^, the smoothness of coating is increased quickly, particularly for chitosan-coated paper. The smoothness of chitosan, DMC and TMC coated paper with the coating weight of 3.0 g/m^2^ is increased 18.2, 6.7 and 5.7 times, respectively, compared to the control (paper substrate). This phenomenon is because chitosan, DMC and TMC solutions with different viscosity fail to evenly spread out over the surface of papers when the coating weight is lower, and then air can seep from cracks on the surface of paper sheets. However, when the coating weight is higher than 1.0 g/m^2^, the surface of paper sheets can be wet by chitosan and N-methylated chitosan and the formed coating is relatively uniform to stop the air flow, which is also can be seen in Fig. 6. Therefore, the smoothness of coated papers is largely increased by coating with TMC.

Scanning electron microscopy (SEM) was employed to study the surface of coated papers with the coating weight of 1.5 g/m^2^. Fig. 6a shows that the surface of fibers is smooth and large number of big holes between fibers on paper substrate. Fig. 6b displays that the surface of chitosan-coated paper is only partly covered and the voids between fibers are decreased in comparison with paper substrate. However, there is more uniform coating on the surface of DMC and TMC-coated papers, and most of the fibers are covered by the coating (Fig. 6c,d). This phenomena are because chitosan solution is hard to form a uniform coating due to its high viscosity, while for DMC and TMC, more hydrogen atoms on amino groups of chitosan were substituted with methyl groups, leading to a better solubility and lower viscosity of N-methylated chitosan^38^. Thus, both DMC and TMC solutions are easier to get a flat and uniform coating, leading to a better warp-resistance and increased strength of paper sheet after coating. In addition, the increased smoothness and more compact surface of paper are beneficial to improve the barrier properties of end products, which is good for the final application in high quality packaging for foods, medicine, or cosmetics, to replace plastics^15^.

## Conclusions

In this work, N-methylated chitosan (i.e. N, N-dimethyl chitosan (DMC) and N, N, N-trimethyl chitosan (TMC)), were successfully prepared via methylation reactions. The chemical structure of DMC and TMC was determined by FTIR and ^1^H NMR, and the degree of substitution of DMC and the degree of quaternization of TMC were calculated to be 91.0% and 5.6%, respectively. Also, in order to improve the physical properties and printability of printing paper, chitosan, DMC, and TMC were used as coating agents to coat paper. Results showed that compared with chitosan and DMC, TMC as a coating agent could significantly improve the mechanical properties of paper sheets due to the higher cationic charge density and better solubility. When the coating weight was 3.0 g/m^2^ (to dry paper), the tensile index, tearing index and burst index of TMC-coated paper increased by 71.5%, 31.7% and 79.1%, respectively, and this increasing rate of tensile index with TMC was clearly higher compared to the typical coating agents (such as chitosan, starch, cellulose nanofibrils) with the comparable coating weight reported in literature. Especially, the wet tensile index of TMC-coated paper increased 4.4 times compared to the control. Moreover, the surface strength, ink absorbency and liquid absorbency of TMC-coated paper were more suitable for offset printing at a higher printing speed. This was because TMC with good solubility could form a more uniform coating on the surface of paper sheet and then increase the bonding strength between fibers via electrostatic interaction. Therefore, TMC as a high-efficient coating agent can be used in the offset process to improve the quality and production efficiency of the printings.

## Materials and Methods

Chitosan (made from α-Chitin, 210 kDa, degree of deacetylation of 84.6%), formaldehyde solution (37 wt.%), formic acid (98 wt.%), NaOH, and acetone were purchased from Sinopharm Chemical Reagent Co., Ltd., China. Dimethyl sulfate was purchased from Linyi Yuanbo Chemical Industry Co., Ltd. Bleached hardwood kraft pulp was a gift from Mudanjiang Hengfeng Paper Co., Ltd., China, and the main components of the pulp were 86.1% of cellulose and 13.9% of hemicelluloses. All materials and chemicals were used without any further modification.

DMC was prepared by the reaction of formaldehyde and chitosan under acidic conditions to form Schiff base via Eschweiler-Clarke reaction^60^. Briefly, 10 g chitosan, 30 g formic acid, 40 g formaldehyde solution, and 180 g deionized water (DI) were added in a three-neck round-bottom flask equipped with a reflux condenser. The solution was heated to 70 °C and stirred magnetically for 118 h. After reduced pressure distillation, 1 mol/L NaOH solution was employed to adjust the pH value to 12 and then a gel was obtained. Subsequently, the gel was washed with DI, and then re-dissolved in acetic acid solution with pH of 4. The solution was dialyzed in DI for one week using regenerated cellulose membrane with a molecular weight cutoff of 8,000–12,000 Da. Finally, DMC was obtained after freeze drying.

The preparation method of TMC using dimethyl sulfate was previously reported by Wu^20^. Ten grams of chitosan was slowly added in 40 mL DI and 160 mL dimethyl sulfate with magnetic stirring and reflux condensing system. Then, 60 g of NaOH and 44 g of NaCl were added in the flask. The solution was heated to 70 °C and a dramatic reaction was observed. After 10 h reaction, the pH value of solution was adjusted to 7, and then dialyzed in DI for one week. Finally, the product of TMC was washed with acetone and then vacuum dried at 50 °C for 1 h.

The obtained samples of chitosan and methylated chitosan were prepared as KBr pellets and scanned in the range of 750–4000 cm^−1^ using a Fourier transform infrared spectrometer (FTIR, Nicolet iS10, USA). The samples of chitosan and methylated chitosan were dissolved in a solution consisting of 10% D_2_O and 90% DCl with the concentration of 10 g/L in a water bath at 80 °C, and ^1^H spectra were recorded on a Nuclear magnetic resonance spectrometer (NMR, Bruker AVANCE III 400 MHz, USA).

Chitosan and its methylated derivatives were dissolved in 2% acetic acid solution with the concentration of 2 mg/mL. The pH value of the solution was adjusted by dipping 1 N NaOH solution, and then the transmittance of the solution at 600 nm was recorded using a UV-vis spectrometer (Hitachi U-4100, Japan). Critical pH value was named as the pH when the transmission rate of the solution is more than 98%, while pH50 was defined as the pH when the transmittance reaches 50%^61^. The tests were conducted at least three times. In addition, the ionic charge density of additives was tested by the method of colloidal titration. This method is based on the stoichiometric combination of positive and negative colloid ions, and the potential change of solution can be measured by a Particle Charge Detector (Mütek BTG PCD-04 Travel, Germany).

Paper sheets with a basis weight of 60 ± 2.5 g/m^2^ were firstly prepared based on ISO 5269-1-79 using a paper sheet forming machine (Xianyang Taist Test Equipment Co., Ltd., ZQJ1-B-II, China). Chitosan and methylated chitosan were dissolved in acetic acid solution (pH = 5.5) with the concentration of 2 wt.%. Then, paper sheets were coated with chitosan and methylated chitosan solution using a wire bar coater (Yeke CCI-1000, China) with various coating weight from 0 to 3.0 g/m^2^. The coated papers were dried at 70 °C for 180 s and conditioned at 23 ± 1 °C and 50 ± 2% relative humidity for 12 h before tests. The tensile index, burst index, tearing index and wet tensile index of the coated papers were measured according to TAPPI standard procedures of T 404 wd-03, T 403 om-02, T 414 om-98, and T 456 om-03, respectively. The smoothness of coated paper with the coating weight of 1.5 g/m^2^ was recorded according to T 479 cm-99 (Bekk Method) using a Smoothness tester (TMI 58-05, USA). Each sample was measured five times for calculating the average value and standard deviations. The surface morphologies of coated papers with the coating weight of 1.5 g/m^2^ were observed using a Scanning Electron Microscope (SEM, Hitachi S-4800, Japan). Before observation, gold spraying was treated on paper sheet samples under vacuum.

The surface strength of coated paper was measured using an IGT printability tester (C1–5, Netherlands) for the simulation of offset printing according to T 499 wd-85. The coating weight of offset papers was 1.5 g/m^2^. The K&N ink was absorbed by samples for 2 min, and then wiped by cotton ball, the reflex factor of green ray of samples was recorded by a reflectance spectrophotometer (X-Rite 528, USA).

Liquid absorbency was also investigated with 0.5 wt.% rhodamine B solution^62^. Rhodamine B was dissolved in anhydrous ethanol and then sealed for storage before use. The solution was coated on the coated paper (size 50 mm × 100 mm) using a cotton brush, and then after10 min, the permeation on the front and back of samples were photographed by a digital camera.

## Data Availability

The data used to support the findings of this study are available from the corresponding author upon request.
